# Ectopic Tooth in Mandibular Canal, Maxillary Sinus, and Mandibular Condyle

**DOI:** 10.1155/2022/3118998

**Published:** 2022-01-22

**Authors:** Mert Akbas, Zülfikar Karabıyık, Altan Varol

**Affiliations:** ^1^Istanbul University, Dentistry Faculty, Department of Oral and Maxillofacial Surgery, Turkey; ^2^Kütahya Health Science University, Dentistry Faculty, Department of Oral and Maxillofacial Surgery, Turkey; ^3^Marmara University, Istanbul, Turkey

## Abstract

Eruption of tooth into place other than tooth-bearing region is defined as ectopic eruption. Although ectopic eruption of tooth is rare, there have been cases in the maxillary sinus, mandibular condyle, nasal cavity, chin, palate, and orbital floor. Due to ectopic teeth's rarity and lack of consensus for ıts treatment, incidence was entitled to be added to literature and discussed. It was reported that odontogenic tumors and cysts can develop around the ectopic tooth. Thus, ectopic teeth can be followed up regularly in case of no signs and symptoms. If the patient has unusual orofacial pain, undiagnosed nasal discharge, maxillary sinusitis, preauricular pain, preauricular fistula, trismus, and lip paresthesia, the patient should be evaluated in terms of ectopic tooth. Whether the patient has signs and symptoms related to ectopic tooth, early intervention for the removal of ectopic tooth along with accompanying lesion is the treatment of choice. Specialists choose intervention way based on their experience. When selecting the intervention, minimally invasive and less morbid way should be preferred. Intraoral approach rather than extraorally should be the first choice to prevent unesthetic scar and damage to facial nerve.

## 1. Introduction

Ectopic tooth is defined as the tooth is not located at dental arch. Some interactions which are not normal during the odontogenesis may cause ectopic eruption. Some factors (iatrogenic, developmental, infection, and genetic) are asserted to cause ectopic eruption [1, 2]. Ectopic tooth can be seen at palate, coronoid process, orbital, nasal septum and cavity, chin and maxillary antrum, condyle, and maxillary sinus [3]. Ectopic tooth can be left asymptomatic during the patients' life. Instead of extraction, they could be followed up regularly because of the fact that lesion and tumor can be developed from ectopic tooth during following period [4]. In rare situations, temporomandibular problems, orofacial pain, result from the ectopic tooth [5–7]. The patient could refer to oral and maxillofacial or otolaryngology clinic with signs and symptoms of facial swelling, pain, nasal obstruction, and sinusitis. In those situations, odontogenic causes should be ruled out.

In this paper, we present 3 ectopic teeth located at mandibular canal, maxillary sinus, and condylar region.

## 2. Case 1

**Figure 1:**
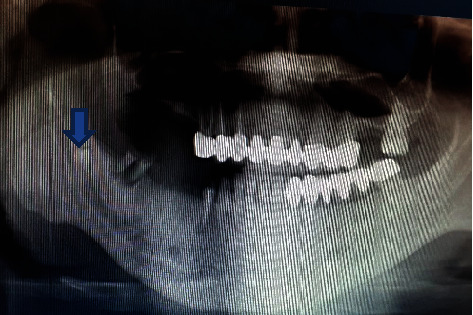
Ectopic premolar tooth seen at OPG.

**Figure 2:**
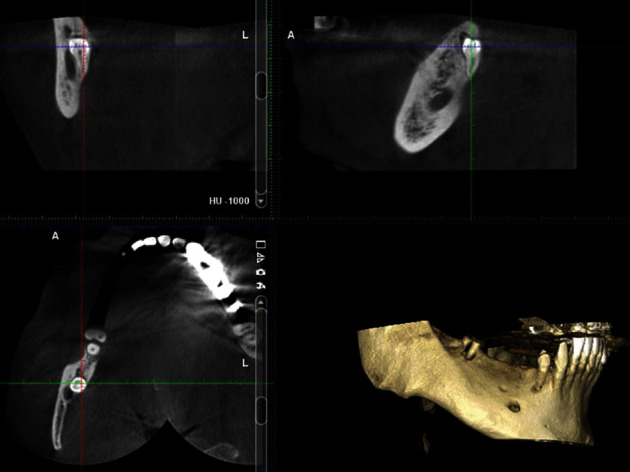
Preoperative CBCT view (coronal, sagittal, axial, and 3D reconstructed section).

A 33-year-old male patient was referred to our clinic with recurred pain at right mandibular region. The patient also had referred pain to his right ear and had paresthesia at region innervated by right nervus alveolaris inferior. Root remnants of right second molar tooth were seen at orthopantomograph (OPG). Pathosis was shown neither OPG nor clinical examination. OPG showed that ectopic premolar tooth superimposed on mandibular canal (Figure 1). Cone beam computed tomography (CBCT) was taken to determine 3D position of ectopic tooth with mandibular canal. CBCT confirmed that ectopic premolar tooth was located in the mandibular canal (Figure 2). Surgery was planned to remove offended ectopic premolar tooth to relieve signs and symptoms.

**Figure 3:**
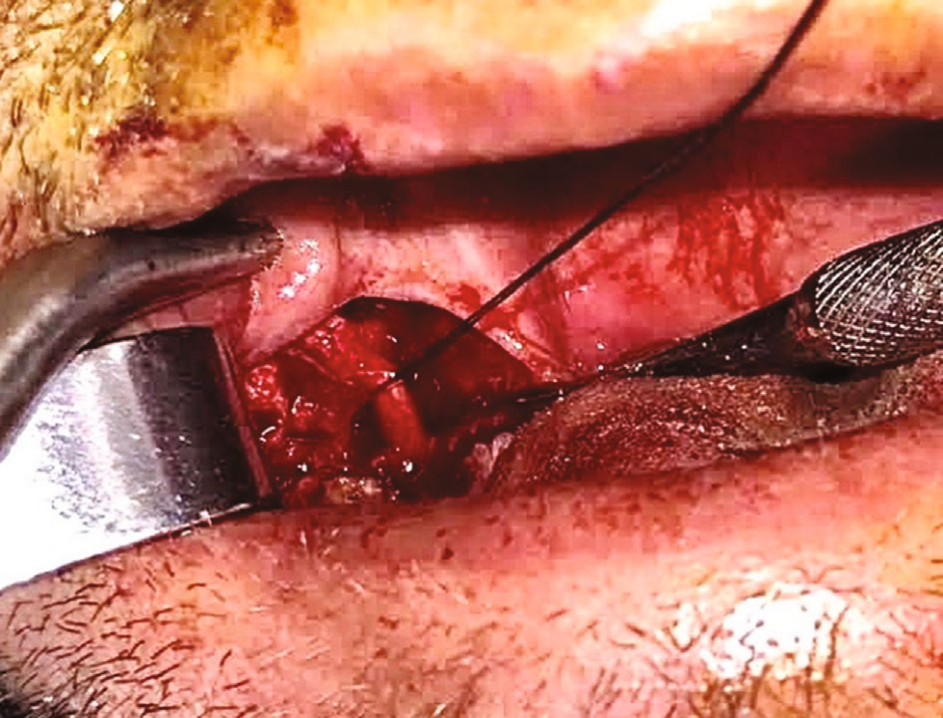
Dissected lingual nerve.

**Figure 4:**
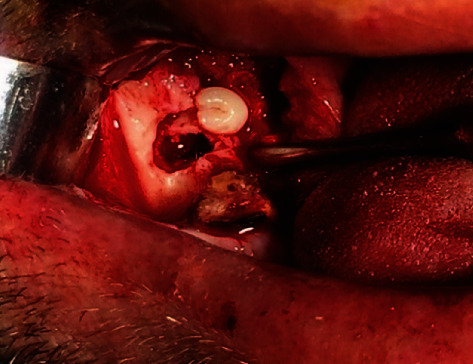
Exposed ectopic wisdom tooth.

**Figure 5:**
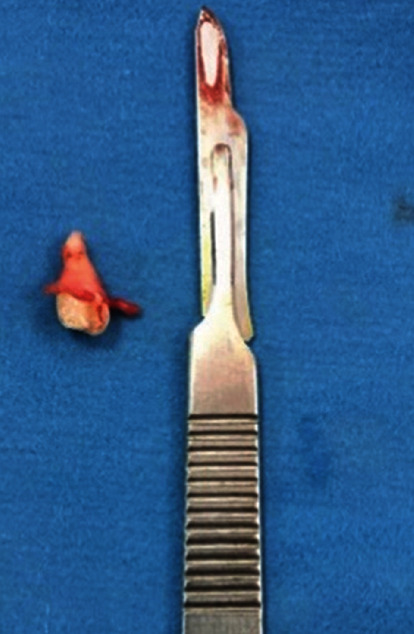
Extracted ectopic premolar tooth and attachment of cyst lining.

**Figure 6:**
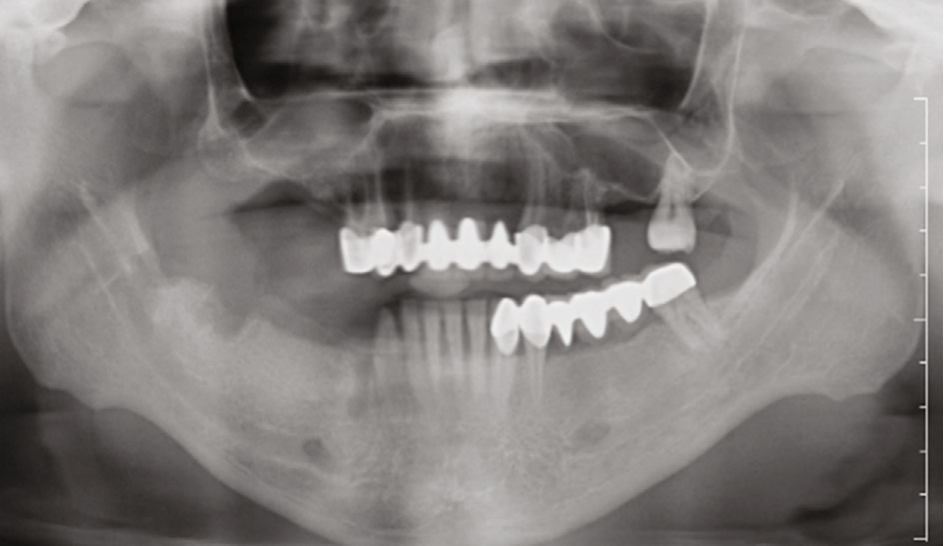
Postoperative orthopantomograph of the patient.

Mucoperiosteal flap was raised, and simultaneously, root remnants of second molar tooth were extracted. Lingual nerve was dissected during the operation by using silk suture (Figure 3). Osteotomy was performed until offended ectopic premolar tooth reached (Figure 4). It was removed at one piece without sectioning (Figure 5). Patient's preoperative symptoms (paresthesia and pain) were disappeared at 6-month follow-up (Figure 6).

## 3. Case 2

**Figure 7:**
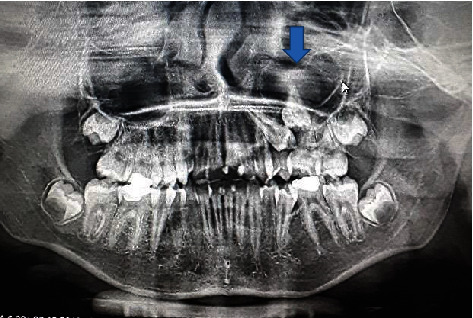
Ectopic second premolar tooth and accompanying lesion.

A 14-year-old male child was referred to dental clinic for orthodontic treatment. Clinical examination of the patient showed absence of upper left second bicuspid. Maxillary left first bicuspid in the nasal floor was planned to be erupted but ectopic tooth (maxillary left second bicuspid) located in the maxillary sinus was removed due to ıts accompanying lesion (radiolucent region surrounding the crown of an ectopic tooth) seen at OPG (Figure 7). Ectopic tooth did not show any signs and symptoms of inflammation. Fluctuation was palpated neither intraorally nor extraorally.

**Figure 8:**
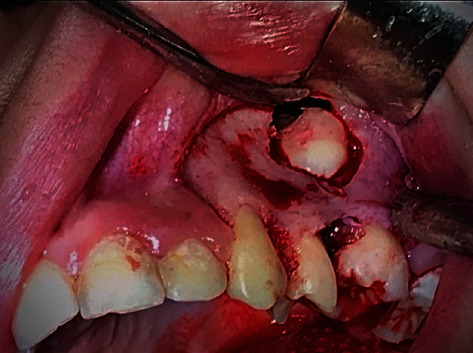
Localization of ectopic tooth.

**Figure 9:**
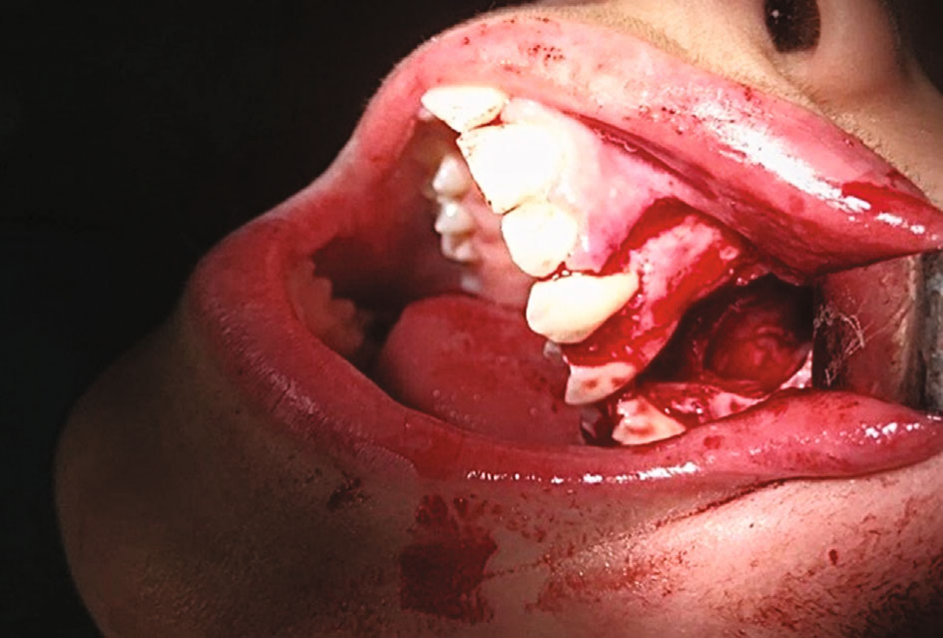
Immediately after the removal of ectopic premolar tooth.

**Figure 10:**
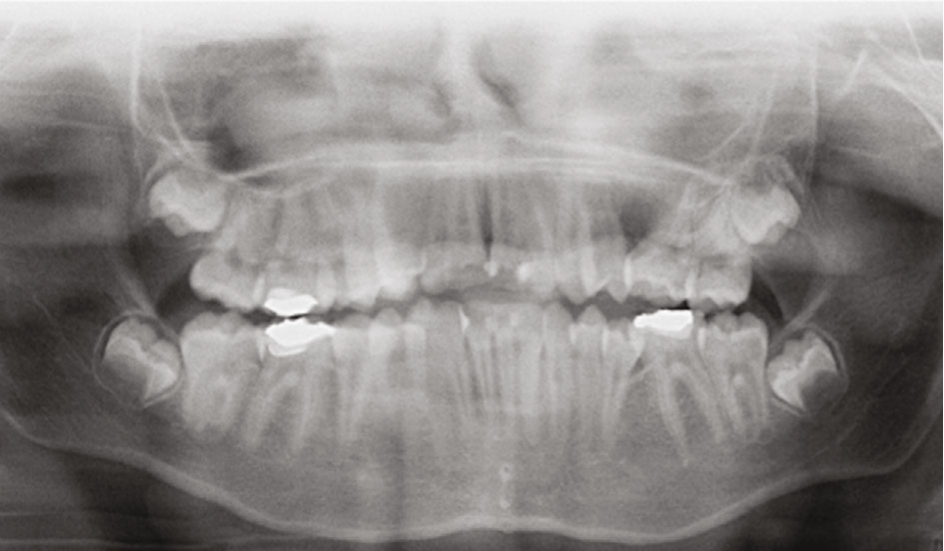
Postoperative 6-month follow-up.

Operation was performed under the local anesthesia, after the mucoperiosteal flap was raised (Figure 8). Caldwell-Luc approach was performed to reach ectopic tooth and ıts associated pathosis (Figure 9). Ectopic tooth and associated pathosis were enucleated. Operated region was irrigated with saline and povidone iodine. Flap was closed primarily. Associated lesion was sent to histopathological examination and verified the dentigerous cyst. The patient was followed up for 1, 3, and 6 months after the operation. Recurrence was not reported, and postoperative period was uneventful (Figure 10).

## 4. Case 3

**Figure 11:**
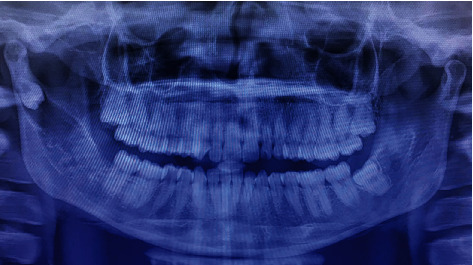
Orthopantomographic view.

**Figure 12:**
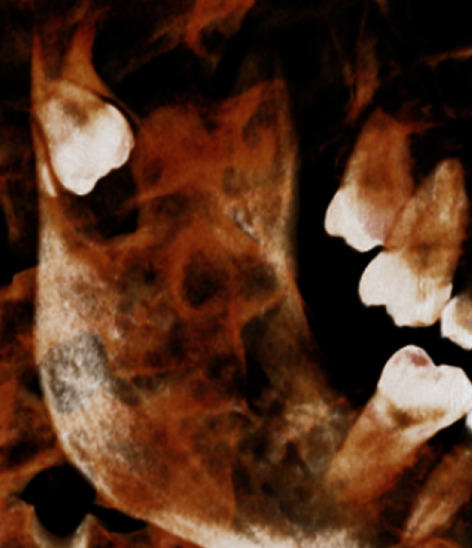
3D reconstructed view.

**Figure 13:**
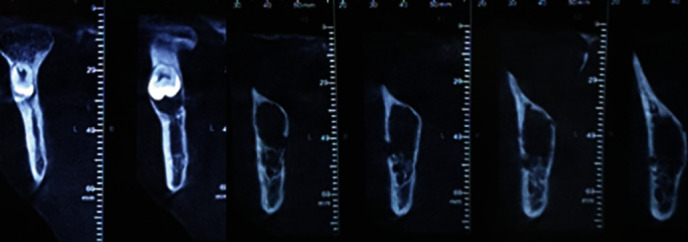
Coronal view.

**Figure 14:**
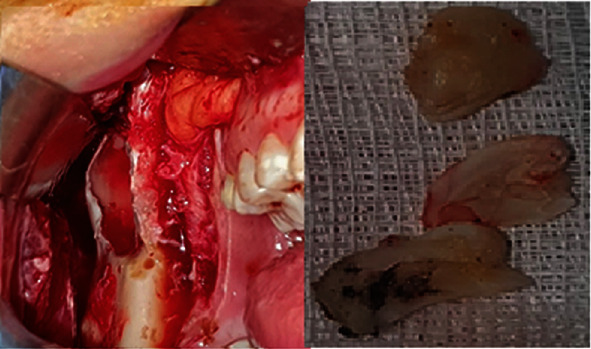
Exposed surgical region and removed tooth (three pieces).

A 28-year-old female patient was referred to dental clinic for dental rehabilitation. Routine OPG was taken (Figure 11). Radiographic assessment was demonstrated at ectopic mandibular third molar positioned in the right condylar region. Radiographically, there was a bony tunnel extending from the third molar to the distal of the second molar. CBCT was obtained to determine the exact position of the ectopic wisdom tooth (Figures 12 and 13). The patient was treated by an intraoral approach on the lateral aspect of the ramus for the removal of the ectopic third molar, as well as tissue in the bony funnel (Figure 14).

Operation was performed under the local anesthesia intraorally to avoid postoperative extraoral scar and damage to facial nerve. Flap was raised to expose coronoid notch and subcondylar region. Osteoectomy was performed to expose ectopic tooth by using high-speed handpiece under the constant irrigation. Ectopic tooth was removed by dividing into 3 pieces to avoid condylar fracture. The soft tissue in the bony canal was completely enucleated. Operated area was rinsed with saline and povidone iodine. It was closed primarily. Soft tissue in the bony canal was sent to histopathological examination and confirmed our prediagnosis as dentigerous cyst. Postoperative period was uneventful. The patient was under the antibiotic coverage and anti-inflammatory analgesic. The patient was followed up regularly. Nerve function was preserved.

## 5. Discussion

Tooth development requires consecutive steps which occur between oral epithelium and mesenchymal tissue. Abnormal interactions during the odontogenesis result in ectopic tooth development. Lower jaw is affected more than upper jaw due to ectopic tooth [8]. Ectopic tooth can not be only permanent but also deciduous and supernumerary tooth [9]. Iatrogenic tooth displacement (tooth germ displacement), pathological cause (cyst and tumor), and developmental abnormalities are asserted factors for the ectopic tooth eruption [10]. In most cases, etiology of ectopic tooth eruption is not determined [4]. In our cases (case 1 and case 3), abnormal position of the tooth germ can be predisposing factor for the ectopic eruption but in case 2, pathosis (tissue surrounding ectopic tooth) can be a factor. Teeth can be erupted at various region such as in sinus maxilla, subcondylar region (as in our cases), nasal floor, chin, orbit, coronoid process, sinus maxilla, and condyle [3, 4].

Dentigerous cysts accompanied to ectopic teeth are reported in the literature [1, 8, 11, 12]. Dentigerous cysts are the second encountered odontogenic cysts of jaws and the most common type of developmental odontogenic cyst of jaws. The pathogenesis of dentigerous cyst is not known but considered factor for ıts development is dental follicle. Fluid accumulation between crown of the tooth and epithelium covering tooth can cause expansion of the dental follicle and result in development of dentigerous cyst [10, 12–14].

A symptomatic or cystic ectopic third molar tooth should be extracted by the intraoral way to avoid damage to facial nerve and scar. Ectopic teeth were associated with pathoses as in our cases (case 2 and case 3). Because of this, they were extracted intraorally. Long-term postoperative follow-up is required until the ossification of mandibular defect is filled with bone tissue when the ectopic tooth is removed from condylar and subcondylar region (case 3). Weakened bone region renders the condyle to pathologic fracture [15].

Although many techniques (nasal endoscopy, transoral endoscopy, and lefort 1 osteotomy) were developed for extracting ectopic tooth from maxillary sinus, Caldwell-Luc procedure is the most common technique while extracting ectopic tooth from maxillary sinus among the oral and maxillofacial specialists (case 2). Complications of Caldwell procedure are to damage of infraorbital nerve, fistula formation, and pain. But operation can be performed in less morbid, invasive way by using endoscope. Selecting the approach to ectopic tooth depends on clinician's experience. Ear, nose, and throat specialists choose mostly endoscope [11].

Teeth can displace into mandible intraosseously. They could not be symptomatic. In those situations, these ectopic teeth can be followed up in a regular basis to rule out cystic or neoplastic changes [16, 17]. Treatment for this type of anomaly varies according to position and conditions. But in the present case (case 1), the patient was symptomatic. The patient had recurrent reflected pain to right ear and paresthesia at lower lip region. In those situations, ectopic tooth should be evaluated.

## 6. Conclusion

Ectopic teeth are rarely seen anomaly in oral and maxillofacial surgery practice. As the patients have unusual pain, swelling, and discharge in maxillofacial region, possibility of the ectopic should be taken into consideration. Asymptomatic ectopic teeth can be followed up regularly because lesions can be developed around such teeth. When extracting the ectopic teeth, minimally invasive and less morbid way should be preferred. Intraoral extraction rather than extraorally should be the first choice because of esthetic outcomes. Patients' life quality should be put in the foreground.
